# Recent Progress in Organic Electrochemical Transistor-Structured Biosensors

**DOI:** 10.3390/bios14070330

**Published:** 2024-07-04

**Authors:** Zhuotao Hu, Yingchao Hu, Lu Huang, Wei Zhong, Jianfeng Zhang, Dengyun Lei, Yayi Chen, Yao Ni, Yuan Liu

**Affiliations:** 1 School of Integrated Circuits, Guangdong University of Technology, Guangzhou 510006, China; hzt18128820581@163.com (Z.H.); 13016055680@163.com (Y.H.); zwnice@163.com (W.Z.); zhangjianfeng@gdut.edu.cn (J.Z.); leidy@gdut.edu.cn (D.L.); chenyy@gdut.edu.cn (Y.C.); 2 School of Physics & Optoelectronic Engineering, Guangdong University of Technology, Guangzhou 510006, China; gz2005hl@163.com

**Keywords:** organic electrochemical transistors, biosensors, electroactive, electro-inactive, cancer cells, sensing systems, wearable and implantable applications

## Abstract

The continued advancement of organic electronic technology will establish organic electrochemical transistors as pivotal instruments in the field of biological detection. Here, we present a comprehensive review of the state-of-the-art technology and advancements in the use of organic electrochemical transistors as biosensors. This review provides an in-depth analysis of the diverse modification materials, methods, and mechanisms utilized in organic electrochemical transistor-structured biosensors (OETBs) for the selective detection of a wide range of target analyte encompassing electroactive species, electro-inactive species, and cancer cells. Recent advances in OETBs for use in sensing systems and wearable and implantable applications are also briefly introduced. Finally, challenges and opportunities in the field are discussed.

## 1. Introduction

Recently, biosensors have experienced vigorous development thanks to the continuous improvement of fabrication techniques for electronic devices [1,2,3]. Biosensors can selectively recognize specific species, leveraging the diversity of biological structures to achieve various functionalities and meet testing requirements [4]. Additionally, they generate corresponding measurement information based on real-time changes in the detected objectives [5], and convert this information into discernible signals according to certain rules for dynamic monitoring [6,7,8,9]. This enables the sensing and detection of biological substances and physiological signals, making them widely applicable in various fields, e.g., healthcare diagnostics, drug screening, food safety, and environmental monitoring [10,11,12,13].

In the realm of biosensing, a multitude of methods have emerged, giving rise to diverse biosensors. Silicon biosensors harness the exceptional properties of silicon materials, enabling the specific recognition and detection of biomolecules through surface modification, and exhibiting advantages including a high sensitivity, repeatability, and integrability [14,15]. Quartz/crystal biosensors capitalize on the unique vibration characteristics of quartz crystals, facilitating biomolecular detection with heightened sensitivity, real-time monitoring, and label-free capabilities [16,17,18]. Glass biosensors exploit the chemical stability and biocompatibility of glass materials, achieving the selective recognition and detection of biomolecules via surface modification or immobilization within the glass matrix [19,20]. Nanomaterial-based biosensors leverage the extensive surface area and distinctive optoelectronic or electrochemical properties of nanomaterials, enabling highly sensitive biomolecule detection [21,22]. Common nanomaterials include gold nanoparticles, carbon nanotubes, and quantum dots [23,24]. Notably, electrochemical biosensors offer both high sensitivity and selectivity, enabling real-time monitoring of biomolecular changes [25,26]. They are characterized by user-friendly operation, portability, and cost-effectiveness, as well as excellent repeatability and stability. Consequently, electrochemical biosensors hold great promise for widespread applications in biotechnology and clinical diagnostics.

According to the working mechanism of the recognition element, biosensors can be classified into two major categories: electroactive biosensors and electro-inactive biosensor [27]. The former utilizes the oxidation–reduction properties of electroactive substances, where the target analyte reacts with the electroactive material on the device’s surface, resulting in changes in current or potential signals [28]; while the latter utilizes bio- electrochemical technique to detect the presence or concentration of a target analyte with bio-recognition methods [29]. These two types of biosensors primarily rely on electrochemical detection methods, which provide a refined and efficient approach for converting biological information into an electrical signal [30,31]. This enables the direct detection of various biological entities with enhanced accuracy and sensitivity [32]. In particular, an electrochemical biosensor with a transistor structure is a classic three-terminal electrochemical biosensing device [33,34]. Its features include a simple structure, rapid response speed, dimensional flexibility, and direct readout of transduction signals [35]. Moreover, its distinct capability to accurately detect low concentrations of analytes under a low operating voltage contributes to its wide applicability and substantial potential for development [36,37].

The rapid development of novel functional materials further brings forth new opportunities for biosensors. Organic materials, renowned for their ease of tailoring, high inherent flexibility, and biocompatibility, have facilitated the design of electrochemical transistor-structured biosensors and have achieved multifunctional applications [38]. The profound coupling ability between ions and electrons exhibited by organic semiconductor active layers contributes significantly to the enhancement of the efficient sensing of microfluidic ions. Organic small molecules offer distinct advantages in terms of high selectivity, sensitivity, and rapid response [39]. Their ability to be precisely designed and synthesized with specific structures enables them to exhibit exceptional selectivity in identifying target molecules while providing exceptional detection limits. However, they are susceptible to environmental conditions, possess limited stability, and involve intricate synthesis and purification processes. On the other hand, conjugated polymers [40] showcase remarkable tunability and superior stability. Through chemical synthesis, their structure and properties can be precisely controlled and fine-tuned to meet diverse sensing requirements. Conjugated polymers commonly exhibit a commendable chemical and physical stability, allowing them to maintain their sensing performance even under varying environmental conditions. Additionally, the sensing layers of conjugated polymers can be rejuvenated or repaired, extending the lifespan of sensors. Furthermore, the deliberate employment of specially designed ion-doped conjugated polymer systems has been proven to accelerate the induction of current, enabling the attainment of highly sensitive signal transduction and precise analysis of biological features [41,42,43]. However, compared with organic small molecules, conjugated polymers may exhibit a relatively lower sensitivity and slower response times. Overall, from the material perspective, major challenges in the development of electrochemical biosensors include, but are not limited to, improving the electrochemical stability of materials, optimizing interfacial transport characteristics, and improving the recognition and selectivity of biomolecules. These challenges need to be addressed in the future by developing novel materials and composite structures, i.e., combining conjugated polymers with small-molecule semiconductors. Improving the mechanical flexibility and biocompatibility of materials has the potential to revolutionize organic electrochemical transistor-structured biosensors (OETBs). These advancements will enable the effective detection of diverse bio-substances which have promising applications in wearable and implantable biosensing systems [44].

**Figure 1:**
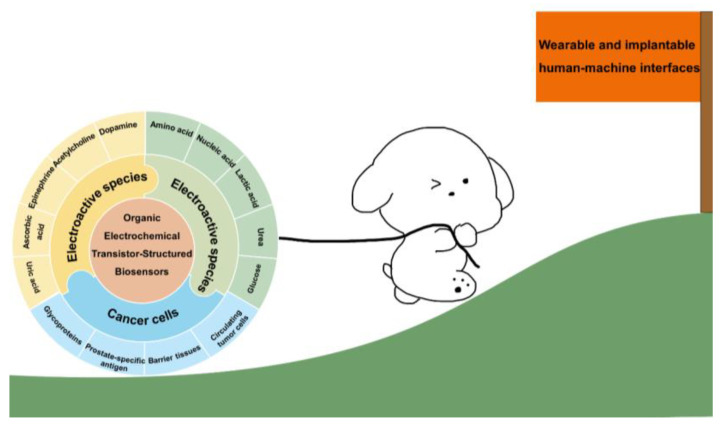
Schematic illustration of the current status and future application of OETBs for species detection.

This article summarizes the technology used in OETBs and their current development status (Figure 1). Section 1 is an introduction; Section 2 presents the basic structure and working principles of OETBs. Section 3, Section 4 and Section 5 detail the OETBs used for detecting electroactive species, electro-inactive species, and cancer cells. Section 6 presents the OETB-constructed wearable and implantable human–machine interfaces. Section 7 summarizes the review and the existing challenges, and suggests paths for further research.

## 2. Basic Structure and Working Principles of OETBs

**Figure 2:**
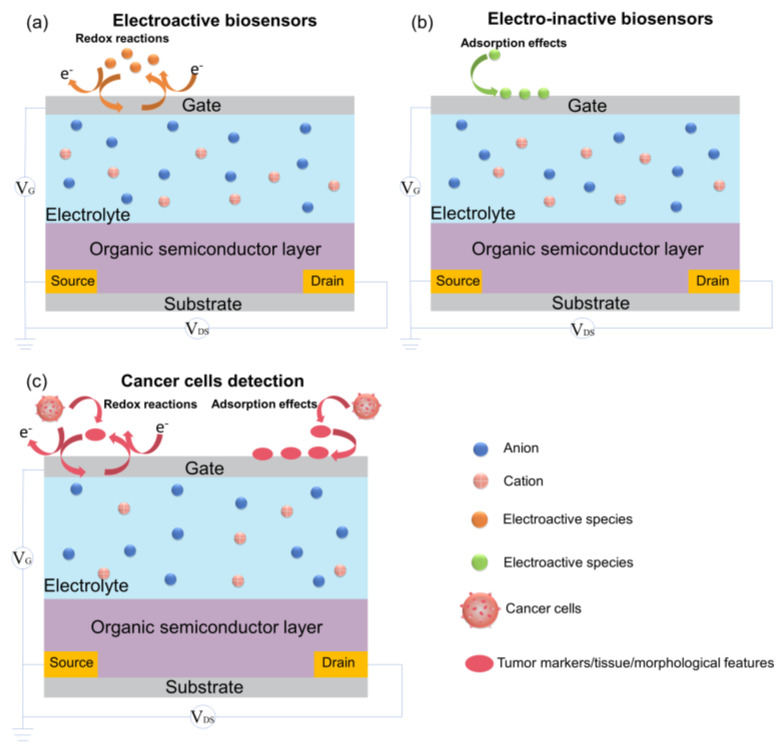
Schematic diagram of the basic structure and working principle of OETBs for the detection of (**a**) electroactive species, (**b**) electro-inactive species, and (**c**) cancer cells.

OETBs share similarities with transistors by consisting of source, drain, and gate electrodes (Figure 2); the gate electrode, which is typically made of metal or carbon materials such as carbon nanotubes or metal oxides [45], is the most important functional electrode for modulating the electric field strength through redox reactions or adsorption effects [46].

Bernard’s model is a classical model used to study the steady state. The most significant difference between OETBs and transistors is that OETBs replace the conventional dielectric layer between the gate electrode and the semiconductor film with an electrolyte [47,48]. When a potential is applied to the gate electrode, ions move into the electrolyte and enter or leave the organic semiconductor layer [44,47,49], resulting in a change in charge density in the organic semiconductor layer, enabling various functions such as rectification and amplification [50,51]. When using liquid electrolytes, the response time usually falls within the range of tens of microseconds, and this minor delay does not significantly affect electrophysiological recording applications. Consequently, for applications that do not require an immediate response, ion gels or solid electrolytes serve as more suitable alternatives [52].

The channel formed between the source and drain electrodes is typically made of a polymer material with high ion conductivity, with PEDOT doped with small anions or poly-anions as dopants, commonly PSS, being the most common [53]. This is mainly because PSS can exist in the form of a water dispersion, allowing for easy deposition on thin films through simple solution processing techniques [54]. The ion transport characteristics of this organic semiconductor layer enable OETBs sensors to respond to changes in biological or chemical molecules [55]. Additionally, diverse techniques for deposition and patterning can be utilized for a range of substrates, such as flexible and stretchable materials. This combination opens up new avenues for creating and designing novel device structures [56].

State and transient signals of OETBs include the ion circuit and electron circuit. In the ion circuit, ions flow from the electrolyte solution into the organic film, altering the doping state and conductivity of the film [36]. The gate voltage *V*_GS_ and drain voltage *V*_DS_ control the injection of ions into the channel, thus regulating the doping state of the semiconductor film. The drain current *I*_DS_ reflects the doping state of the channel, which is directly proportional to the number of mobile charge carriers in the channel. In the electron circuit, charges flow through the path between the source, channel, and drain electrodes. The ion flow is represented as the sum of the contact resistance and channel resistance [57], while the ion volume in the channel is treated as a capacitance [58]. Various factors, such as channel thickness, ion–electron mixed transport, and bilayer coupling of conjugated polymers, influence the performance of OETBs.

## 3. OETBs for Electroactive Species Detection

Electroactive species, including neurotransmitters, vitamins, antioxidants, and some electroactive metabolites, undergo electrochemical redox reactions at the functionalized working interface of biosensors. These reactions result in a change in current that is directly proportional to the concentration of the analyte. The concentration-dependent oxidation current density enables the direct application of electrochemical transducer systems for the detection of electroactive metabolites.

### 3.1. Neurotransmitters Detection

**Figure 3:**
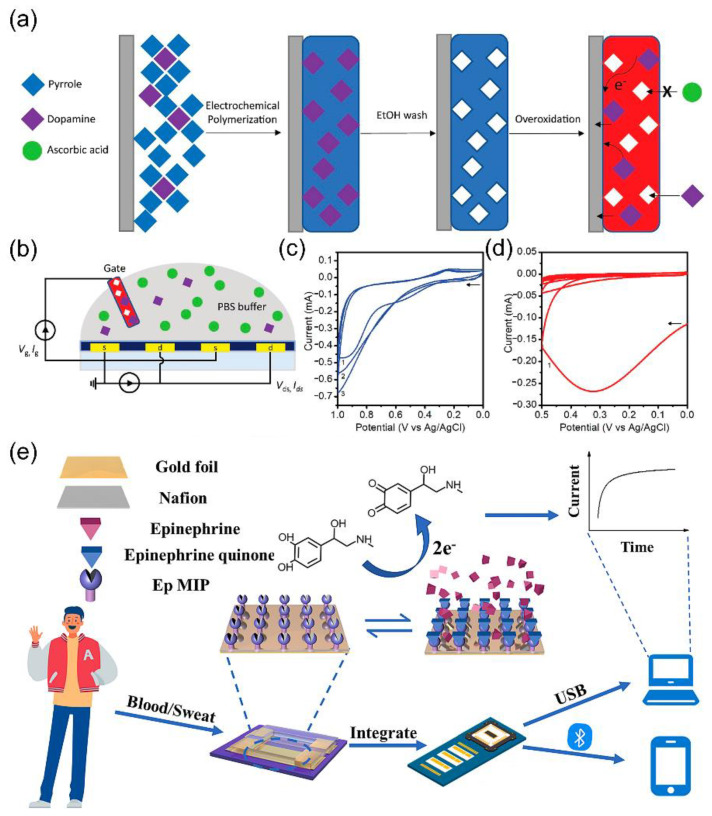
(**a**) Electrochemical synthesis of dopamine-specific o-molecular imprinted polymer (o-MIP) on Pt electrode. (**b**) Schematic representation of the OETB with overoxidized dopamine-specific MIP as the gate electrode. (**c**) Cyclic voltammogram of the pyrrole (10 mM) polymerization with the presence of dopamine template (1 mM) on Pt electrode. (**d**) Cyclic voltammogram of the overoxidation of polypyrrole MIP in 0.5 M NaOH [60]. Copyright from 2022, American Chemical Society. (**e**) Schematic illustration of the epinephrine OETB design and operational principle [69]. Copyright from 2023, Elsevier.

Dopamine plays a vital role as one of the primary neurotransmitters in the human body, and its concentrations in bodily fluids are typically very low, ranging from the pM to μM levels [59]. Electroactive OETBs are widely used for sensing dopamine and rely on the gate voltage offset caused by redox chemistry at the electrode. Tang et al. [60] developed a highly sensitive dopamine OETB and compared the efficacy of various gate electrodes, e.g., gold (Au) and platinum (Pt) electrodes, to optimize the sensor’s performance (Figure 3a–d). The results showed that the sensor achieved an impressive detection limit for dopamine, reaching approximately 5 nM. However, the simultaneous presence of ascorbic acid and uric acid alongside dopamine in the body poses a challenge to the use of traditional electrochemical methods, as the responses of these three compounds often exhibit a significant overlap. Despite the high catalytic activity and conductivity exhibited by gate electrodes composed of traditional precious metals, such as gate electrodes made of Au and Pt, their electrocatalytic behavior typically lacks selectivity towards dopamine.

Depositing a modification layer on the electrode can effectively enhance the selectivity of dopamine detection. By featuring an alkaline over-oxidized molecularly imprinted polymer polypyrrole on the Pt gate, the OETB demonstrated a high selectivity with a signal ratio between the target and interfering substances greater than five in the concentration range of 400 nM to 10 μM, regardless of when the interference was introduced, while the sensor with a bare Pt gate exhibited almost no selectivity towards dopamine.

Given the poor toxicity resistance, low natural abundance, and high cost of noble metals, the development of precious metal-free gate electrodes has gradually garnered attention. OETBs fabricated utilizing a Nafion and reduced graphene oxide-wrapped carbonized silk fabric (Nafion/rGO/CSF) as a composite gate electrode exhibit excellent dopamine sensing capabilities, including high selectivity and sensitivity [61]. The hierarchical structure of the composite electrode derived from natural silk fabric can effectively prevent the aggregation of rGO and Nafion, enhancing the conductivity and amplifying the electrochemical properties. This configuration enables the OETB sensor to achieve an exceptionally low detection limit of 1 nM for DA, with a wide detection range from 1 nM to 30 μM. Similarly, nitrogen/oxygen-codoped carbon cloths, serving as precious metal-free gate electrodes, also demonstrate distinct electrochemical sensing behaviors towards ascorbic acid and dopamine [62]. This presents a novel avenue for the design of OETBs with exceptional sensitivity and selectivity.

Compared to conventional thin-film electrodes, fiber-shaped gate electrodes may exhibit superior performance, characterized by improved current amplification and response rates. For instance, when a polyamide 6 filament was integrated with PVA-*co*-PE nanofibers and a polypyrrole nanofiber network, the resulting OETBs demonstrated an enhanced response time, stability, and reproducibility [63]. The three-dimensional nanofiber network served as an effective platform, enabling superior sensitivity even with dopamine concentrations as low as 1 nM, along with excellent selectivity in the presence of interferents.

Epinephrine, a neurotransmitter and hormone, plays a vital role in many instinctive responses [64,65]. Non-invasive and biocompatible detection methods are highly desirable for monitoring epinephrine levels in the human body. Coppede et al. [66,67] developed an integrated system with two OETBs on cotton fibers for non-invasive sensing of epinephrine in human sweat. One OETB used a silver (Ag) wire as the gate electrode, while the other employed a Pt wire. The device, based on poly(3,4-ethylene dioxythiophene)–poly(styrene sulfonate) PEDOT–PSS-functionalized cotton yarn and Pt wire, detected epinephrine concentrations above 1 μM through oxidation at the platinum gate, resulting in color changes.

To enhance the sensitivity of epinephrine detection in OETBs, electrode modifications using low-cost carbon-based nanomaterials like graphene flakes, graphene oxide, and single-walled carbon nanotubes (CNTs) have been explored [68]. In particular, incorporating Nafion/nanomaterial-based modifications into Pt gate electrodes has proven to be highly effective. These modified OETBs have demonstrated exceptional performances, achieving an impressive detection limit of 100 pM. This advancement in electrode modification holds great promise for highly sensitive and cost-effective epinephrine sensing in OETBs. The gate electrode modified with a molecular imprinting polymer also enables the recognition of epinephrine molecules through the voltage drop caused by redox reactions (Figure 3e) [69]. This type of OETB has a detection limit of as low as 10 pM, making it suitable for detecting humoral metabolites. It can be used for at least 20 cycles of detection and can be easily recovered through a simple positive voltage cleaning process, which is cost effective for wearable devices.

**Figure 4:**
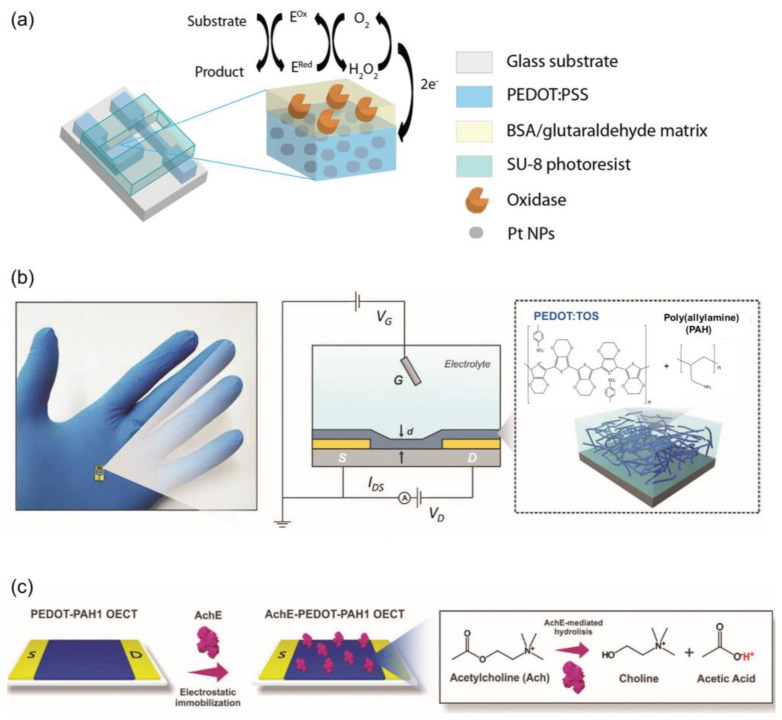
(**a**) Schematic diagram of the OETB neurotransmitter sensor and the enzymatic sensing mechanism [72]. Copyright from 2014, Wiley-VCH. (**b**) Scheme showing an OETB, the measurement setup used and a representation of the polyelectrolyte-conducting polymer blend. (**c**) Scheme of the electrostatic immobilization strategy and the enzyme-catalyzed Ach hydrolysis [73]. Copyright from 2021, Wiley-VCH.

Acetylcholine plays a crucial role in the central nervous system and supports learning, memory, and muscle activity in the peripheral nervous system [70,71]. Detecting local acetylcholine concentrations with a high sensitivity and specificity is important. Studies have focused on the enzymatic detection of acetylcholine using bulk platinum as the gate material. By incorporating nanomaterials like CNTs and Pt nanoparticles (Pt NPs) into the gate electrode, its sensitivity and detection limits have been improved. Kergoat et al. (Figure 4a) developed selective and sensitive OETBs by using Pt NPs in PEDOT–PSS electrodes, showing a sensitivity of 4.1 mol/L·cm^2^ for acetylcholine [72].

OETBs can be constructed using a composite material, specifically poly(3,4-ethylenedioxythiophene)–tosylate or polyallylamine hydrochloride, as the conducting channel in Au electrodes, which offer an ideal solution for the detection of acetylcholine. By adjusting the ratio of polyelectrolyte to conducting polymer, the electronic and ionic properties of the transistors can be easily controlled (Figure 4b,c) [73]. The integration of pH-sensitive amino groups further enhances their performance: the immobilization of acetylcholinesterase using NH_2_ groups enables the OETBs to detect acetylcholine within a range of 5–125 µm.

### 3.2. Vitamins and Antioxidants Category Detection

**Figure 5:**
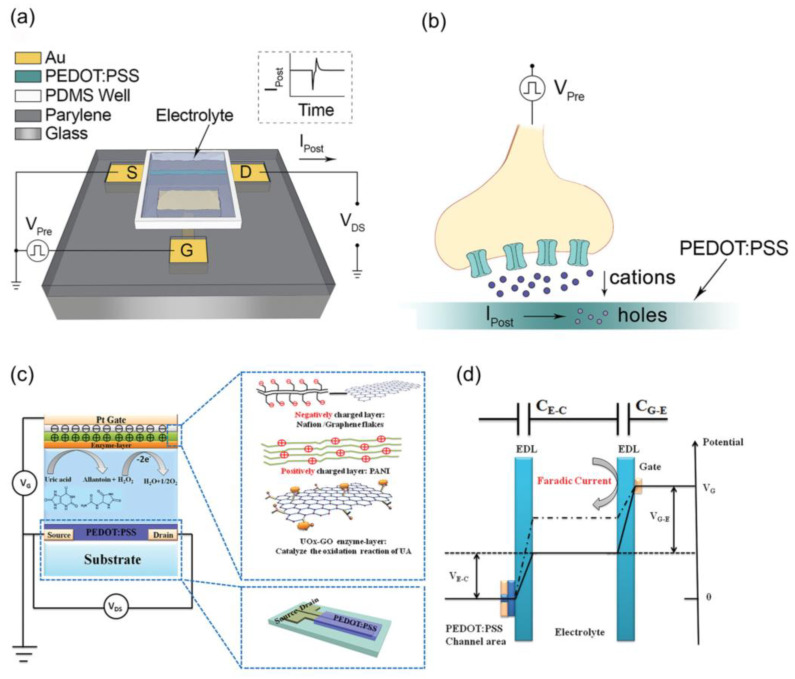
(**a**) OETB schematic and wiring diagram. (**b**) Schematic representation of the synaptic OETB in analogy to a biological synapse [75]. Copyright from 2015, Wiley-VCH. (**c**) Schematic diagram of an OETB with an enzyme uricase-graphene oxide (UO_x_–GO)/polyaniline (PANI)/Nafion-graphene/Pt gate. (**d**) Potential drops between the gate and channel of the OETB before (solid line) and after (dash line) the addition of UA in the electrolyte (PBS solution) [76]. Copyright from 2015, Wiley-VCH.

In addition to the aforementioned neurotransmitters, substances in the vitamins and antioxidants category are also applicable for detection using electroactive OETBs. Ascorbic acid, a crucial biological compound, plays a key role in various metabolic reactions [74]. The high susceptibility of ascorbic acid to oxidation makes the electroactive OETBs suitable devices for its detection. Among the materials employed as electrode modifiers, conducting polymers are widely used. By adjusting the concentration of oxidized sites within the detection potential range, the charge carriers in the channel can be effectively regulated. A typical PEDOT–PSS polymer with unique deposition techniques offers a cost-effective alternative to expensive metal gate electrodes (Figure 5a,b), significantly reducing manufacturing costs [75]. Due to its excellent mechanical properties, PEDOT–PSS is an ideal electrode choice for flexible OETBs specifically designed for ascorbic acid monitoring. Upon ascorbic acid interaction, PEDOT–PSS facilitates the extraction of charge carriers from the channel, resulting in a decrease in current. Under optimal conditions, the OETBs have a remarkable detection limit of 10 nM and an impressive sensitivity of 4.5 μA per decade.

The development of highly flexible and anti-biofouling OETBs based on polymer materials shows potential for the long-term monitoring of ascorbic acid in the body [77]. These OETBs, composed of fiber-shaped PEDOT–PSS on a fluorine rubber insulating layer, offer inherent flexibility, preventing tissue damage and inflammation caused by device micro-movements. Additionally, the hydrophilic nature of PEDOT–PSS ensures resistance to biofouling, maintaining a high sensitivity despite protein adsorption that hampers chemical substance diffusion and compromises accuracy. The all-polymer fiber-OETBs achieved a sensitivity of 0.587 mA per decade and provided stable 14-day monitoring of ascorbic acid in the mouse brain.

The precise recognition of ascorbic acid is an additional crucial parameter for OETBs. Using molecularly imprinted polymers created through the molecular imprinting technique is an effective method for enhancing the selectivity and applicability of OETBs [78]. The molecularly imprinted polymer-constructed OETBs demonstrate an impressive detection limit of 10 nM with a signal-to-noise ratio (S/N) of >3 and a sensitivity of 75.3 μA per decade. Importantly, these sensors exhibit exceptional specificity in recognizing ascorbic acid, effectively eliminating interference from structurally similar compounds, common metal ions, and other substances.

### 3.3. Electroactive Metabolites Detection

Some of the metabolites of organic acids can participate in electrochemical reactions under appropriate conditions, making them suitable for electroactivity detection. Uric acid is one of the byproducts of purine metabolism in the human body [79,80]. Its basal concentrations in physiological samples can vary significantly, typically ranging from 100 nM to 1 mM. To address the need for accurate detection of uric acid, OETBs with Pt gate electrodes and active layers composed of EDOT–PSS have emerged as a leading solution (Figure 5c,d) [76]. These devices have demonstrated excellent selectivity and a low detection limit of only 10 nM, which is approximately three orders of magnitude better than conventional electrochemical methods that use the same enzyme electrodes.

In the context of uric acid monitoring in wound exudate, a textile-based OETB configuration utilizing poly PEDOT–PSS has been developed [81]. This configuration has proven to reliably and reversibly detect uric acid concentrations in synthetic wound exudate within the biologically relevant range of 220–750 μM. Moreover, it has shown great potential in non-invasive detection applications in the human body.

Molecular imprinting polymer-enhanced OETBs could selectively recognize uric acid from various interfering substances. Tao et al. [82] utilized a molecular imprinting polymer film-modified gate electrode to create OETBs based on PEDOT/rGO/cotton fibers. The introduction of rGO increased the specific surface area of the fibers, resulting in the formation of fibrous nanoclusters composed of numerous interconnected nanowires of PEDOT. As a result, the fabricated sensor demonstrated the highest sensitivity of 100 μA and normalized current response per decade for uric acid concentrations ranging from 1 nM to 500 μM.

Sialic acid is commonly found at the terminal end of cell surface glycoproteins and glycolipids [83]. OETBs utilizing gate electrodes modified with multi-walled CNTs and covalently bound 3-aminophenylboronic acid demonstrate specific recognition of sialic acid [84]. By precisely controlling the gate voltage, these OETBs generate adaptive source-drain channel current signals, enabling the sensitive detection of sialic acid across a range of 0.1 to 7 mM concentrations.

## 4. OETBs for Electro-Inactive Species Detection

In contrast to the detection of electroactive substances, electrochemical biosensing of inactive species relies on a cascade process that integrates bio-recognition with physicochemical signal transduction [85]. This involves utilizing biomolecular labels (e.g., enzymes) and artificial mediators (e.g., molecularly imprinted polymer) to indirectly convert the biological signal into an electrochemical signal for identification [2]. Electro-inactive species, in this context, typically refer to biomolecules and electro-inactive metabolites which are targeted for detection and analysis in biosensing applications [86].

### 4.1. Electro-Inactive Metabolites Detection

**Figure 6:**
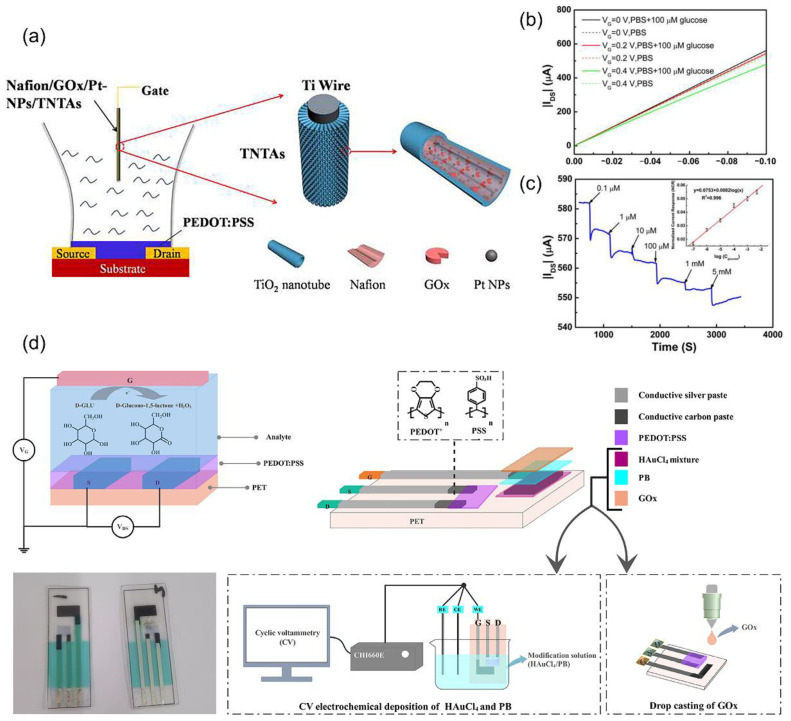
(**a**) The schematic diagram of the device structure of PEDOT–PSS based OETB glucose sensor using Nafion/glucose oxidase (GOx)/Pt nanoparticles (Pt-NPs)/TiO_2_ nanotube arrays (TNTAs) as a gate electrode. (**b**) The output characteristic of an OETB with the Nafion/GOx/Pt-NPs/TNTAs gate electrode measured in PBS solution with and without containing glucose. (**c**) The current response of the OETB device to successive additions of glucose. The inset shows the normalized current response (NCR) as a function of glucose concentration [90]. Copyright from 2015, SAGE Publications Inc. (**d**) Electrodes were prepared according to the OETB principle. The conductive tracks were made using conductive silver paste and carbon paste. Using the electrochemical workstation, HAuCl_4_ mixture and PB solution were deposited into the gate by the CV method, and GO_x_ was attached to the gate by the drop casting method to produce a multi-layer modified sensor [91]. Copyright from 2024, Elsevier.

The regulation of glucose metabolism is indicative of an individual’s level of health [87,88]. The utilization of Pt NPs and glucose oxidase-modified electrodes demonstrates a remarkable sensitivity to glucose. These modified electrodes exhibit a linear response to the logarithm of glucose concentration within the range of 100 nM to 5 mM, with an impressive detection limit as low as 100 nM [89]. Furthermore, when the gate electrode is enhanced with Nafion modification, selective detection of glucose becomes possible even in the presence of interfering substances such as ascorbic acid and uric acid, e.g., the gate electrode of an OETB can be modified with biocompatible polymers such as chitosan and Nafion, as well as graphene or rGO, along with glucose oxidase (GOx) (Figure 6a–c) [90]. The incorporation of chitosan and Nafion aids in the immobilization of GOx on the gate electrode, thereby enhancing the selectivity of the device. Meanwhile, graphene and rGO serve to augment the charge transfer and surface area of the gate electrode, subsequently improving the device’s sensitivity. Consequently, the detection limit can be extended to a range of 10 nM to 1 μM.

Moreover, by incorporating multiple layers of Au film, Prussian blue, and glucose oxidase into the gate electrode, and employing a PEDOT–PSS film to cover the channel between the source and drain electrodes, an OETB becomes capable of accurately identifying glucose levels in sweat (Figure 6d) [91]. This device achieves an impressive low detection limit of 0.10 μM, even when faced with interfering substances such as uric acid, dopamine, urea, sodium chloride, and ascorbic acid.

An accurate quantification of urea is crucial for medical diagnosis, particularly in assessing kidney and liver dysfunction [92,93]. A highly sensitive method for urea detection involves utilizing OETBs in conjunction with specific enzymes. The enzymes facilitate the conversion of the interaction between urea analyte and enzymes into an electronic signal, enabling precise detection. Various strategies have been explored to achieve this goal. For instance, one approach involves integrating urease into OETBs based on graphene or reduced graphene oxide [76]. These biosensors exhibit a wide detection range from 1 μM to 1 mM, providing a comprehensive span for urea measurement. Another method involves confining urease within a gelatin hydrogel, which acts as a bridge between the PEDOT–PSS gate electrode and channel. Urease, as a catalyst, facilitates the hydrolysis of urea into ammonium ions and bicarbonate ions [94]. The presence of ammonium ions induces a de-doping effect on the PEDOT–PSS channel, resulting in a decrease in drain current. This change in current serves as the basis for urea detection. The biosensor demonstrates an impressive detection limit of 1 μM and exhibits a rapid response within approximately 2–3 min across a wide dynamic range.

**Figure 7:**
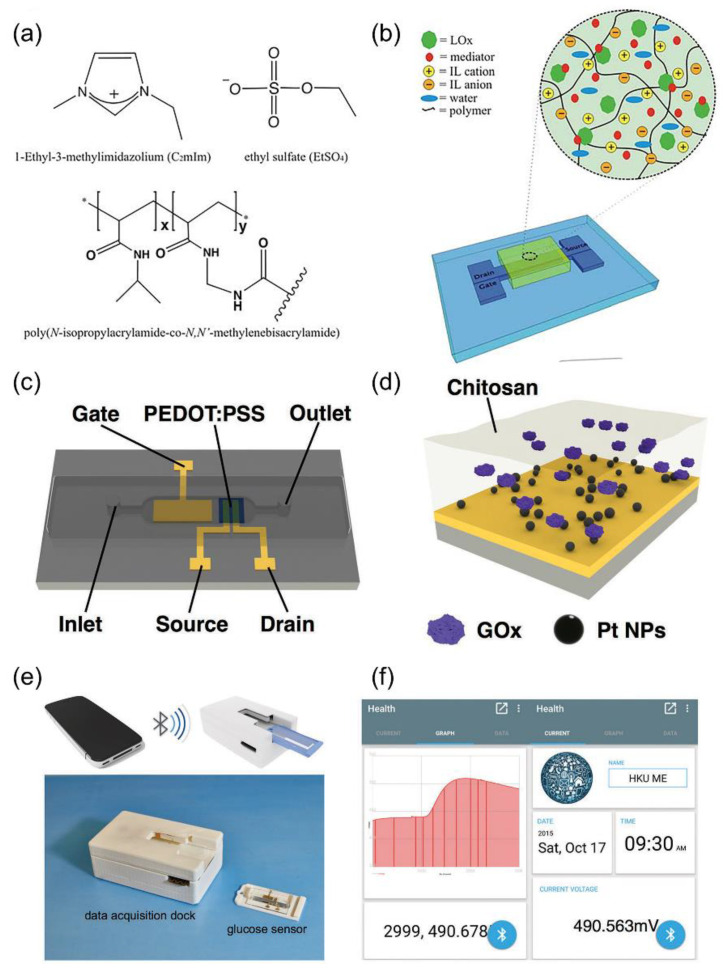
(**a**) Ion gel components and (**b**) a schematic representation of the OETB device with ion gel/enzyme mixture [99]. Copyright from 2012, Springer. (**c**) Schematic diagram of glucose sensor based on OETB integrated with microfluidic channel. (**d**) Gate electrode modification of device. (**e**) Schematic diagram of portable glucose sensor that can interact with smartphone. (**f**) Left side is main interface of app. Right side is detection curve of glucose. *X*-axis represents the time scale (0.1 s of one count), *Y*-axis represents the real-time voltage measured [101]. Copyright from 2016, Wiley-VCH.

Lactic acid, a crucial marker of tissue oxygenation, is a relevant indicator of physiological response during exercise [95,96]. Lactate, the deprotonated form of lactic acid in sweat with a concentration range of 9–23 mM, serves as an indicator for indirectly measuring lactic acid levels [97,98]. An ion gel can be utilized to create a biosensor hat that is both solid state and capable of analyzing the levels of lactate in sweat (Figure 7a,b) [99]. This technology enables the development of lactic acid biosensors using printed OETBs with an organically modified ion gel. In this design, the mediator is covalently bound and the enzyme is entrapped within the sensor. The OETBs have been successfully validated for detecting lactate in human sweat, with a detection limit of 3.7 mM [100]. The sensitivity of OETBs can be enhanced by modifying the gate electrode with lactate oxidase and poly(n-vinyl-2-pyrrolidone)-capped Pt NPs, replacing the conventional bulk electrode. This modification allows for a detection limit of lactate as low as 1 μM (Figure 7c–f) [101].

### 4.2. Biomolecules Detection

The widespread application of OETBs for the detection of electro-inactive species has been hindered by the limited availability of enzymes and selective membranes, as well as their susceptibility to deactivation [30,33]. By leveraging specific interactions between analytes and modified substances, significant improvements can be achieved in the efficiency of detecting electro-inactive species. An effective approach involves the use of molecularly imprinted polymer films as a modification layer [102,103], enabling selective and electrocatalytic oxidation of target molecules such as tryptophan (Trp) and tyrosine (Tyr). When combined with the amplification capability of the transistor, this integration results in highly sensitive OETBs, which exhibit a linear response range of 300 nM to 10 μM for L-Trp and L-Tyr, with sensitivities of 3.19 and 3.64 μA/μM and detection limits of 2 nM for L-Trp and 30 nM for L-Tyr (S/N > 3) [104].

Similarly, histidine (His) can be effectively detected utilizing a molecularly imprinted polymer strategy. Within the concentration range of 100 nM to 10 μM, the modulation of OETB channel current exhibits a direct proportionality to the levels of both bioactive L-His and biologically inactive D-His. The detection limits for L-His and D-His are determined to be 10 nM and 100 nM, respectively, satisfying a S/N exceeding three. Notably, the channel current responses and interferences between L-His and D-His demonstrate discernible disparities, underscoring the exceptional selectivity of the molecularly imprinted polymer film [105].

Nucleic acid diagnostics holds significant research value across various fields, including gene expression monitoring and viral and bacterial identification [106,107,108]. Traditional OETBs with a high sensitivity primarily rely on materials like carbon nanotubes, graphene, and poly-electrolyte multilayers, which involve complex synthesis and modification steps. Alternative approaches using DNA amplification have been developed to address these challenges. Signal amplification strategies such as loop-mediated amplification and hybridization chain reaction (HCR) have been established [109]. Integrating HCR-based nucleic acid self-assembly into OETBs enables the creation of long DNA biosensors from small amounts of target material. Gold nanoparticles are electrochemically deposited on the gold gate electrode to increase surface area, facilitating the connection of HCR products (long negatively charged double-stranded DNA) to the target material through hybridization. This enhances the effective gate voltage offset of OETBs, resulting in a high sensitivity for detecting target DNA with concentrations as low as 0.1 pM. Moreover, such sensors exhibit excellent selectivity, capable of distinguishing target DNA from mismatched DNA.

Integrating photochemically active gate electrodes enhances the sensitivity of DNA biosensing in OETBs. The interaction between photocurrent and the photosensitive material on the electrode enables the operation of photochemical OETBs, making them responsive to biorecognition events. This approach utilizes independent signals for photoexcitation and electrochemical detection, effectively reducing background noise and achieving a high sensitivity with low detection limits [110]. OETBs incorporating PEDOT–PSS as the active layer, CdS quantum dots (QDs) modified ITO glass as the photosensitive layer, and a gate electrode in a phosphate-buffered saline solution with 0.1 M ascorbic acid as the electrolyte, have demonstrated the ability to detect target DNA concentrations as low as 1 fM. This superior performance is attributed to the efficient absorption of higher-energy photons by CdS QDs, causing electron excitation and the formation of electron–hole pairs. The presence of ascorbic acid as a strong electron donor effectively prevents electron–hole pair recombination, leading to the stabilization of conduction band electrons on the ITO gate electrode. Single-stranded DNA is immobilized on the CdS QDs, enabling the capture of target DNA labeled with Au NPs. The interaction between excitons in CdS QDs and plasmons in Au NPs influences charge transfer on the gate electrode, resulting in amplified signal output.

## 5. OETBs for Cancer Cells Detection

The electrochemical detection of cancer cells typically involves the identification of metabolites and tumor markers, as well as the tissue and morphological features associated with cancer cells [111,112,113]. Cancer cell metabolism often leads to the generation or release of specific metabolites [114,115]. Tumor markers are specific molecules that are produced and released into bodily fluids during cancer development [116]. Morphological characteristics refer to the irregular and atypical features observed in cancer cells compared to normal cells, as well as the formation of structures with specific compositions [117,118]. The previously mentioned cancer cell metabolites, e.g., lactate and sialic acid, will not be further expanded upon in this discussion. The focus of this discussion is on tumor markers and their tissue and morphological features. 

### 5.1. Tumor Markers Detection

Proteins serve as commonly utilized cancer markers in the field of oncology [119,120]. However, traditional protein sensors may lack the required sensitivity when detecting certain biomarkers with extremely low concentrations in physiological environments due to the typically weak interactions between proteins and organic semiconductors. OETBs can address this limitation by providing an enhanced sensitivity through their inherent amplification capability. These OETBs demonstrate an ability to specifically detect cancer markers, e.g., human epidermal growth factor receptor 2, with a detection limit as low as 10 fg mL^−1^ [121].

**Figure 8:**
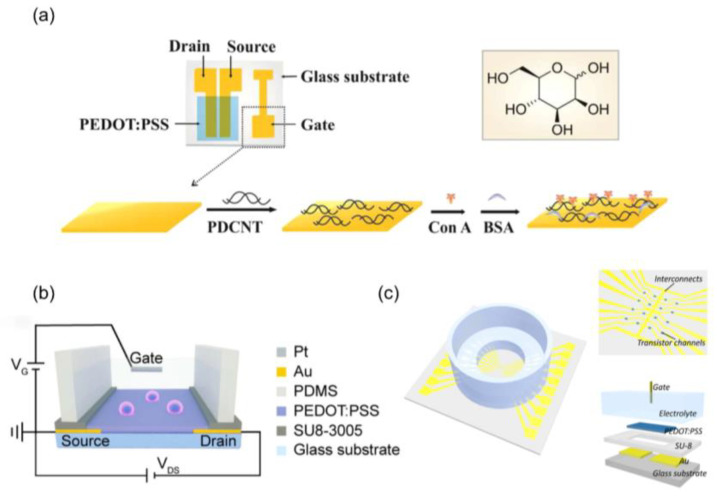
(**a**) Design illustration of the OETBs for glycan sensing. Inset: chemical structure of mannose [125]. Copyright from 2012, American Association for Cancer Research Inc. (**b**) The schematic diagram of a cell sensor based on a solution gated OETB [126]. Copyright from 2023, Royal Society of Chemistry. (**c**) Configuration of the assembled 16-chanbel OETB platform. The OETB array composing of Au electrodes, SU-8 passivation and PEDOT: PSS channel was fabricated on a glass substrate. The gate electrode was immersed into the electrolyte to give bias [127]. Copyright from 2019, SAGE Publications Inc.

Glycoproteins, as protein–sugar complexes, are essential components found on cell surfaces in eukaryotic cells, playing key roles in cancer progression [122,123,124]. A specially designed OETB, incorporating a PEDOT–PSS channel and a gate electrode modified with poly dimethyl diallyl ammonium chloride/multi-walled CNTs has been proven to effectively and specifically bind mannose and detect glycan molecules onto the surface of cancer cells (Figure 8a) [125]. The OETB generates direct current signals that are influenced by the mannose content on the cell surfaces, thereby impacting the captured cell count. It exhibits significant current responses even at low concentrations of cancer cells (i.e., 10 cells/μL). Moreover, by modifying the lectin and aptamer sequences, the OETB can be adapted for the analysis of various glycans and cancer cells.

Prostate-specific antigen, a serine protease regulated by androgens, is widely used as a serum biomarker for preoperative diagnosis and screening of prostate cancer [128,129,130]. The impressive sensitivity and expanded dynamic range of the OETBs were achieved by leveraging the signal amplification effect of Au NPs. The OETBs comprising PEDOT: PSS and conjugated with Au NPs linked to secondary antibodies, exhibited a remarkable detection limit of 1 pg/mL for the prostate-specific antigen-α1-antichymotrypsin complex [131].

Circulating tumor cells have emerged as valuable tumor biomarkers in liquid biopsy, as they originate from primary tumors and enter the circulatory system [132,133]. A specialized OETB based on PEDOT–PSS channel and poly(dimethylsiloxane) (PDMS) reservoir can detect these circulating tumor cells (Figure 8b) [126]. It utilizes chemically conjugated specific antibodies on the conductive film surface to selectively capture tumor cells. By monitoring the effective gate voltage potential shift, the number of captured cells on the conductive film can be determined, enabling accurate cell detection and high-throughput screening. For instance, when the concentration of cancer cells reached 5000, the captured cells completely covered the PEDOT–PSS surface, resulting in an observed effective gate voltage shift of approximately 63 mV.

### 5.2. Tissue and Morphological Features Detection

Barrier tissues, e.g., epithelial cells, are vital for preventing the infiltration of circulating tumor cells, which is a key characteristic of malignancy [134,135]. Evaluating barrier function is crucial in cancer research [136]. OETBs composed of a glass substrate, metal interconnects, and a PEDOT–PSS can be utilized to assess barrier function. A thin protective layer of SU-8 on the PEDOT–PSS active region directly comes into contact with deposited cells and the culture medium (Figure 8c) [127]. By employing frequency-dependent transconductance measurements, epithelial cell lines and cancer cell lines can be distinguished based on their unique morphologies and Transepithelial Electrical Resistance values. This differentiation offers valuable insights into the spatial invasion of cancer cells into the normal epithelium.

An OETB fabricated using a solution-gated carboxyl graphene and PEDOT–PSS mixture has a remarkable capability to detect cancer cell metabolism and monitor changes in cancer cell morphology in situ [137]. By incorporating carboxyl graphene into PEDOT–PSS with concentrations ranging from 0 to 5 mg/mL, the surface area of cancer cells increased from 218 μm^2^ to 530 μm^2^. Furthermore, the OETB exhibits the ability to capture and analyze various changes in cell morphology. The electronegativity of cancer cells triggers a noticeable positive adjustment in the gate voltage, typically around 40 mV for cells with a spherical configuration. Concurrently, as the surface area of the unit expands, the transfer characteristics manifest a distinct negative shift in the gate voltage. This method yields a fresh insight into the detection of cancer cells.

## 6. OETBs for Wearable and Implantable Human-Machine Interfaces

OETBs serve as signal amplification components and offer practicality and ease of miniaturization; meanwhile, they exhibit excellent compatibility with biology and electronics [138,139], making them particularly suitable for bio-electronic interfaces [140,141,142]. To achieve good interfaces in OETBs, several factors need to be considered. First and foremost is material selection, with a focus on biocompatible and flexible/stretchable materials [143]. This ensures the device’s compatibility with human tissue, improves comfort, and enhances durability [144]. Additionally, signal transmission and processing play a crucial role. Attention should be given to electrical signal transmission methods and data processing algorithms to effectively analyze and interpret biological signals obtained from the body. Another significant aspect is energy supply and management. OETBs can be powered by in vivo or in vitro energy-harvesting techniques like biofuel cells or wireless charging technology [145]. It is also important to design low-power circuits to prolong the device’s operational lifespan [146]. In terms of application scenarios, OETBs have the potential to continuously monitor vital signs [147], blood sugar levels [148], and provide treatments such as nerve stimulation for chronic pain or Parkinson’s disease [149]. Security and privacy considerations are also vital, ensuring the confidentiality of patient data and the device’s reliability even under extreme conditions [150]. In addition, by carefully selecting and optimizing materials, designing efficient device layouts, and considering energy supply and management, OETBs can be further enhanced for various bio-electronic interface applications. Specifically, carbon cloth gate electrodes, paper-based flexible structures [151], textile-based wearable forms, wearable patches, and microfluidics-integrated configurations are employed to develop these bio-signal monitoring systems using OETBs [152,153,154]. A typical study employed a simple template-free electropolymerization process to design OETBs featuring nanostructured poly(3,4-Ethylenedioxythiophene) derivatives in the channel layer for detecting cortisol in sweat [155]. Such a nanostructure facilitates the immobilization of cortisol antibodies on the polymer layer. The developed cortisol immune-sensing system demonstrated linear detection in the concentration range of 1 fg/mL to 1 μg/mL, with a low limit of detection of 0.0088 fg/mL. It exhibited excellent stability, repeatability, and shelf life. Consequently, when tested with cortisol added to artificial sweat, this system exhibited a strong practical performance in clinical environments and wearable sensors.

Bio-inspired transistor-like devices serve as the core of human–machine interfaces and enable integrated sensing and computation of multimodal signals [156,157,158]. They have found extensive applications in the field of bio-monitoring systems [159,160,161,162,163]. One prominent example is the development of an electrophysiological signal monitoring system for brain activity based on OETBs. This system enables early clinical diagnosis, computed tomography scan examinations, perioperative predictions, patient physiological assessment, and surgical guidance. By combining biodegradable materials with an integration approach for 100-level channel neural interfaces, this system ensures mm-scale assembly of neural interfaces for cellular-level mapping. It provides a stable high transconductance of up to 9.0 mS and enhances electrophysiological signals with a signal-to-noise ratio of up to 37 dB in practical use. In animal experiments, it can distinguish between normal and abnormal regions using active matrix recording technology. After completing its task, the system can self-decompose, eliminating the need for further surgical retrieval. This multi-channel transient OETBs platform can seamlessly laminate onto the cerebral cortex, laying a solid foundation for the capabilities of implantable transient electronic technologies in various biomedical applications.

An overview of the major biosensors in terms of detection object, modification, transconductance (g_m_), gain, limit of detection (LOD), and time response is given in Table 1.

## 7. Conclusions

Organic electronic biosensors serve as a cornerstone in the field of bioelectronics, aiming to enhance existing biomedical technologies through their primary connectivity with biological constituents. This article elucidates the operational principles and recent advancements of OETBs, with a specific emphasis on applications in human chemical substance detection, cancer cell detection, and neurological applications.

OETBs demonstrate superiority over traditional electrochemical methods and other generic devices. Primarily, they exhibit stability across diverse electrolytes, such as cerebrospinal fluid, cellular media, sweat, and blood. Moreover, OETBs showcase high transconductance values and gain properties, significantly enhancing detection sensitivity. The time response can be further improved by adjusting the geometric configuration and dimensions of the channels. Additionally, OETBs seamlessly integrate with biological systems, including individual cells, tissues, or even entire organs, facilitated by diverse molecular modifications of gate electrodes and conductive polymers.

However, the development of OETBs faces noteworthy challenges. These encompass the exploration and implementation of novel active materials possessing improved conductivity, stability, patterning, and reabsorbability. Furthermore, there is an urgent need to develop OETBs that are compatible with photolithography techniques, enabling the integration of sensors with multi-scale microarrays and the incorporation of circuits for power supply, recording, and transmission.

Overall, OETBs hold tremendous potential for advancing tissue engineering within the human body. With the evolution of the Internet of Things and big data technologies, wearable devices utilizing organic chemical sensors are envisioned to foster point-of-care health monitoring, thereby facilitating life-saving interventions and disease prevention through inter-device communication. By amalgamating physical sensor technologies, wearable chemical sensors are poised to deliver valuable diagnostic data for human health monitoring and future nanobiotechnology endeavors.

## Figures and Tables

**Table 1:** An overview of the major biosensors in terms of detection object, modification, gm, gain, LOD, and time response.

Detection Object	Modification	g_m_	Gain	LOD	Time Response	Ref.
Dopamine	o-MIP/Pt	~0.11 mS	~5.5	34 nM	-	[60]
	Nafion/rGO/CSF	~40 mS	~1.2	1 nM	-	[61]
	NOCCs	~20 mS	~4.15	0.17 µM	-	[62]
	FECTs	~17 mS	~2.42	1 nM	0.34 s	[63]
Epinephrine	Cotton-OECT	~10 mS	~2.36	1 µM	<1 s	[66]
	Nafion and SWNTs	~3.1 mS	~1.2	0.1 nM	-	[68]
	MIP	~30 mS	~3	10 pM	-	[69]
Acetylcholine	PEDOT–PSS/Pt NPs	~0.06 mS	~20	5 µM	-	[72]
	PEDOT–TOS	~41.7 mS	-	5 µm	-	[73]
Ascorbic acid	PEDOT–PSS	-	-	0.1 µM	-	[75]
	PF-OECT	~2.73 mS	~3.5	10 µM	-	[77]
	MIP	~17 mS	~3.5	10 nM	-	[78]
Uric acid	PANI/Nafion	~6 mS	~1.5	0.1 µM	-	[76]
	PEDOT–PSS	~4 mS	~3.5	220 µM	400 s	[81]
	MIP	15.5 mS	~2.3	1 nM	-	[82]
Sialic acid	Carbon Nanotubes	0.9 mS	~1.5	0.1 mM	-	[83]
Glucose	rGO and enzyme	~5.08 mS	~1.1	10 nM	-	[89]
	TNTAs	~5.83 mS	~1.1	100 nM	-	[90]
	Prussian Blue	~2.6 mS	~1.3	0.1 µM	-	[91]
Urea	PET	~1.83 mS	~4.7	1 µM	2–3 min	[94]
Lactic acid	Iongel	-	-	10 mM	-	[99]
	Pt NPs	~6.5 mS	~1.9	1 µM	1 min	[101]
Trp Tyr	MIP	~17 mS	~8	2 nM	-	[104]
D-His & L-His	MIP	~13.5 mS	~1.2	10 nM	-	[105]
DNA	HCR	~2 mS	~2	0.1 pM	-	[109]
	QDs	~3.5 mS	~7	1 fM	-	[110]
Tumor Proteins	Catalytic nano-probes.	~5.3 mS	-	10 fg mL^−1^	-	[121]
Tumor Glycoproteins	Nano-probes	~2.7 mS	-	10 cells/µL	-	[125]
Tumor Prostate-specific antigen	AuNPs–PSA pAb	~36 mS	-	1 pg/ml	-	[131]
Cortisol	anostructured poly(3,4-Ethylenedioxythiophene) derivatives	-	-	0.0088 fg/mL	-	[155]

## Data Availability

Not applicable.

## References

[1] Naresh V., Lee N. (2021). A Review on Biosensors and Recent Development of Nanostructured Materials-Enabled Biosensors. Sensors.

[2] Singh A., Sharma A., Ahmed A., Sundramoorthy A.K., Furukawa H., Arya S., Khosla A. (2021). Recent Advances in Electrochemical Biosensors: Applications, Challenges, and Future Scope. Biosensors.

[3] Sardini E., Serpelloni M., Tonello S. (2020). Printed Electrochemical Biosensors: Opportunities and Metrological Challenges. Biosensors.

[4] Agius F., González-Lamothe R., Caballero J., Muñoz-Blanco J., Botella M., Valpuesta V. (2003). Engineering Increased Vitamin C Levels in Plants by Overexpression of a D-galacturonic Acid Reductase. Nat. Biotechnol..

[5] Wang Q., Zhao C., Sun Y., Xu R., Li C., Wang C., Liu W., Gu J., Shi Y., Yang L. (2023). Synaptic Transistor with Multiple Biological Functions Based on Metal-organic Frameworks Combined with the LIF Model of a Spiking Neural Network to Recognize Temporal Information. Microsyst. Nanoeng..

[6] Potje-Kamloth K. (2008). Semiconductor Junction Gas Sensors. Chem. Rev..

[7] Someya T., Dodabalapur A., Huang J., See K.C., Katz H.E. (2010). Chemical and Physical Sensing by Organic Field-Effect Transistors and Related Devices. Adv. Mater..

[8] Huang J.L., Miragliotta J., Becknell A., Katz H.E. (2007). Hydroxy-terminated Organic Semiconductor-based Field-effect Transistors for Phosphonate Vapor Detection. J. Am. Chem. Soc..

[9] Yu H., Wang T., Li Y., Luo P., Ni Y. (2023). Organic Synaptic Transistors with an Ultra-Short-Term Weight-Reconstruction for Processing Multiple Types of Signals. Adv. Electron. Mater..

[10] Ni Y., Feng J., Liu J., Yu H., Wei H., Du Y., Liu L., Sun L., Zhou J., Xu W. (2022). An Artificial Nerve Capable of UV-Perception, NIR–Vis Switchable Plasticity Modulation, and Motion State Monitoring. Adv. Sci..

[11] Pan X., Zhang Z., Su M. (2024). Colloidal self-assembly in biosensing strategies for biomarkers diagnosis. Innov. Mater..

[12] Truong P.L., Yin Y., Lee D., Ko S.H. (2023). Advancement in COVID-19 Detection Using Nanomaterial-based Biosensors. Exploration.

[13] Wang J., Chen D., Huang W., Yang N., Yuan Q., Yang Y. (2023). Aptamer-functionalized Field-effect Tansistor Biosensors for Disease Diagnosis and Environmental Monitoring. Exploration.

[14] Clechet P., Jaffrezic-Renault N. (1990). Silica Surface Sensitization and Chemical Sensors. Adv. Mater..

[15] Harraz F.A. (2014). Porous Silicon Chemical Sensors and Biosensors: A review. Sens. Actuators B Chem..

[16] Lim H.J., Saha T., Tey B.T., Tan W.S., Ooi C.W. (2020). Quartz Crystal Microbalance-based Biosensors as Rapid Diagnostic Devices for Infectious Diseases. Biosens. Bioelectron..

[17] Park H.J., Lee S.S. (2018). A Quartz Crystal Microbalance-based Biosensor for Enzymatic Detection of Hemoglobin A1c in Whole Blood. Sens. Actuators B Chem..

[18] Alanazi N., Almutairi M., Alodhayb A. (2023). A Review of Quartz Crystal Microbalance for Chemical and Biological Sensing Applications. Sens. Imaging.

[19] Maas M.B., Maybery G.H.C., Perold W.J. (2018). Borosilicate Glass Fiber-Optic Biosensor for the Detection of Escherichia coli. Curr. Microbiol..

[20] Barrio M., Cases R., Cebolla V., Hirsch T., Marcos S., Wilhelm S., Galbán J. (2016). A Reagentless Enzymatic Fluorescent Biosensor for Glucose based on Upconverting Glasses, as Excitation Source, and Chemically Modified Glucose Oxidase. Talanta.

[21] Su H., Li S., Jin Y., Xian Z., Yang D., Zhou W., Kerman K. (2017). Nanomaterial-based Biosensors for Biological Detections. Adv. Health Care Technol..

[22] Lan L., Yao Y., Ping J., Ying Y. (2017). Recent Advances in Nanomaterial-based Biosensors for Antibiotics Detection. Biosens. Bioelectron..

[23] Yun Y.-H., Eteshola E., Bhattacharya A., Dong Z., Shim J.-S., Conforti L., Kim D., Schulz M.J., Ahn C.H., Watts N. (2009). Tiny Medicine: Nanomaterial-Based Biosensors. Sensors.

[24] Lee S.H., Sung J.H., Park T.H. (2012). Nanomaterial-Based Biosensor as an Emerging Tool for Biomedical Applications. Ann. Biomed. Eng..

[25] Kurnik M., Pang E.Z., Plaxco K.W. (2020). An Electrochemical Biosensor Architecture Based on Protein Folding Supports Direct Real-Time Measurements in Whole Blood. Angew. Chem. Int. Ed..

[26] Campuzano S., Yáñez-Sedeño P., Pingarrón J.M. (2019). Reagentless and Reusable Electrochemical Affinity Biosensors for Near Real-time and/or Continuous Operation. Advances and Prospects. Curr. Opin. Electrochem..

[27] Bai L., Elósegui C.G., Li W., Yu P., Fei J., Mao L. (2019). Biological Applications of Organic Electrochemical Transistors: Electrochemical Biosensors and Electrophysiology Recording. Front. Chem..

[28] Wallace G.G., Smyth M., Zhao H. (1999). Conducting Electroactive Polymer-based Biosensors, Trac-Tend. Anal. Chem..

[29] Strakosas X., Bongo M., Owens R.M. (2015). The Organic Electrochemical Transistor for Biological Applications. J. Appl. Polym. Sci..

[30] Rivnay J., Inal S., Salleo A., Owens R.M., Berggren M., Malliaras G.G. (2018). Organic Electrochemical Transistors. Nat. Rev. Mater..

[31] Liu Z., Zhao Y., Yin Z. (2024). Low-power Soft Transistors Triggering Revolutionary Electronics. Innovation.

[32] Nawaz A., Liu Q., Leong W.L., Fairfull-Smith K.E., Sonar P. (2021). Organic Electrochemical Transistors for in Vivo Bioelectronics. Adv. Mater..

[33] Marks A., Griggs S., Gasparini N., Moser M. (2022). Organic Electrochemical Transistors: An Emerging Technology for Biosensing. Adv. Mater. Interfaces.

[34] Friedlein J.T., McLeod R.R., Rivnay J. (2018). Device physorctics of organic electrochemical transistors. Org. Electron..

[35] Sophocleous M., Contat-Rodrigo L., García-Breijo E., Georgiou J. (2021). Organic Electrochemical Transistors as an Emerging Platform for Bio-Sensing Applications: A Review. IEEE Sens. J..

[36] Rashid R.B., Ji X., Rivnay J. (2021). Organic Electrochemical Transistors in Bioelectronic Circuits. Biosens. Bioelectron..

[37] Ravariu C. (2023). From Enzymatic Dopamine Biosensors to OECT Biosensors of Dopamine. Biosensors.

[38] Shen H., Di C.A., Zhu D. (2017). Organic transistor for bioelectronic applications. Sci. China-Chem..

[39] Afreen S., Muthoosamy K., Manickam S., Hashim U. (2015). Functionalized fullerene (C60) as a Potential Nanomediator in the Fabrication of Highly Sensitive Biosensors. Biosens. Bioelectron..

[40] Moczko E., Istamboulie G., Calas-Blanchard C., Rouillon R., Noguer T. (2012). Biosensor Employing Screen-printed PEDOT: PSS for Sensitive Detection of Phenolic Compounds in Water. J. Polym. Sci. Part A Polym. Chem..

[41] Kergoat L., Piro B., Berggren M. (2012). Advances in Organic Transistor-based Biosensors: From Organic Electrochemical Transistors to Electrolyte-gated Organic Field-effect Transistors. Anal. Bioanal. Chem..

[42] Bernards D.A., Malliaras G.G. (2007). Steady-State and Transient Behavior of Organic Electrochemical Transistors. Adv. Funct. Mater..

[43] Romele P., Gkoupidenis P., Koutsouras D.A. (2020). Multiscale Real Time and High Sensitivity Ion Detection with Complementary Organic Electrochemical Transistors Amplifier. Nat. Commun..

[44] Kukhta N.A., Marks A., Luscombe C.K. (2022). Molecular Design Strategies toward Improvement of Charge Injection and Ionic Conduction in Organic Mixed Ionic–Electronic Conductors for Organic Electrochemical Transistors. Chem. Soc. Rev..

[45] Tian X., Liu D., Bai J., Chan K.S., Ip L.C., Chan P.K.L., Zhang S. (2022). Pushing OECTs toward Wearable: Development of a Miniaturized Analytical Control Unit for Wireless Device Characterization. Anal. Chem..

[46] Yu S., Sun X., Liu J., Li S. (2024). OECT-Inspired Electrical Detection. Talanta.

[47] Zhang P., Zhu B., Du P., Travas-Sejdic J. (2024). Electrochemical and Electrical Biosensors for Wearable and Implantable Electronics Based on Conducting Polymers and Carbon-Based Materials. Chem. Soc. Rev..

[48] Sun H., Gerasimov J., Berggren M., Fabiano S. (2018). N-Type Organic Electrochemical Transistors: Materials and Challenges. J. Mater. Chem..

[49] Li X., Feng Y., Shi L., Zhou J., Ni Y. (2024). Far-gate Synaptic Transistors Utilizing Ion-charge Dual-transfer Mechanism for Neurotransmitter-multiplexing Temporal Coding. Appl. Phys. Lett..

[50] Wu X., Chen S., Moser M., Moudgil A., Griggs S., Marks A., Li T., McCulloch I., Leong W.L. (2023). High Performing Solid-state Organic Electrochemical Transistors Enabled by Glycolated Polythiophene and Ion-gel electrolyte with a Wide Operation Temperature Range From −50 to 110 °C. Adv. Funct. Mater..

[51] Bischak C.G., Flagg L.Q., Ginger D.S. (2020). Ion Exchange Gels Allow Organic Electrochemical Transistor Operation with Hydrophobic Polymers in Aqueous Solution. Adv. Mater..

[52] Chen S., Hou K., Li T., Wu X., Wang Z., Wei L., Leong W.L. (2023). Ultra-lightweight, Highly Permeable, and Waterproof Fibrous Organic Electrochemical Transistors for on-skin Bioelectronics. Adv. Mater. Technol..

[53] Shi H., Liu C., Jiang Q., Xu J. (2015). Effective Approaches to Improve the Electrical Conductivity of PEDOT: PSS: A Review. Adv. Electron. Mater..

[54] Kayser L.V., Lipomi D.J. (2019). Stretchable Conductive Polymers and Composites Based on PEDOT and PEDOT: PSS. Adv. Mater..

[55] Fan X., Nie W., Tsai H., Wang N., Huang H., Cheng Y., Wen R., Ma L., YaN F., Xia Y. (2019). PEDOT: PSS for Flexible and Stretchable Electronics: Modifications, Strategies, and Applications. Adv. Sci..

[56] Donahue M.J., Sanchez-Sanchez A., Inal S., Qu J., Owens R.M., Mecerreyes D., Malliaras G.G., Martin D.C. (2020). Tailoring PEDOT Properties for Applications in Bioelectronics. Mater. Sci. Eng. R Rep..

[57] Nissa J., Janson P., Berggren M., Simon D.T. (2021). The Role of Relative Capacitances in Impedance Sensing with Organic Electrochemical Transistors. Adv. Electron. Mater..

[58] Friedlein J.T., Rivnay J., Dunlap D.H., McCulloch I., Shaheen S.E., McLeod R.R., Malliaras G.G. (2017). Influence of Disorder on Transfer Characteristics of Organic Electrochemical Transistors. Appl. Phys. Lett..

[59] Klein M.O., Battagello D.S., Cardoso A.R., Hauser D.N., Bittencourt J.C., Correa R.G. (2019). Dopamine: Functions, Signaling, and Association with Neurological Diseases. Cell. Mol. Neurobiol..

[60] Tang K., Turner C., Case L., Mehrehjedy A., He X., Miao W., Guo S. (2022). Organic Electrochemical Transistor with Molecularly Imprinted Polymer-Modified Gate for the Real-Time Selective Detection of Dopamine. ACS Appl. Polym. Mater..

[61] Ji W., Wu D., Tang W., Xi X., Su Y., Guo X., Liu R. (2020). Carbonized Silk Fabric-based Flexible Organic Electrochemical Transistors for Highly Sensitive and Selective Dopamine Detection. Sens. Actuators B Chem..

[62] Xi X., Wu D., Ji W., Zhang S., Tang W., Su Y., Guo X., Liu R. (2020). Manipulating the Sensitivity and Selectivity of OECT-Based Biosensors via the Surface Engineering of Carbon Cloth Gate Electrodes. Adv. Funct. Mater..

[63] Qing X., Wang Y., Zhang Y., Ding X., Zhong W., Wang D., Wang W., Liu Q., Liu K., Li M. (2019). Wearable Fiber-Based Organic Electrochemical Transistors as a Platform for Highly Sensitive Dopamine Monitoring. ACS Appl. Mater. Interfaces.

[64] Wortsman J. (2002). Role of epinephrine in acute stress. Endocrinol. Metab. Clin. North Am..

[65] Wong D.L. (2006). Epinephrine Biosynthesis: Hormonal and Neural Control During Stress. Cell. Mol. Neurobiol..

[66] Coppedè N., Tarabella G., Villani M., Calestani D., Iannotta S., Zappettini A. (2014). Human stress monitoring through an organic cotton-fiber biosensor. J. Mater. Chem. B.

[67] Coppedè N., Ferrara L., Bifulco P., Villani M., Iannotta S., Zappettini A., Cesarelli M., Fabrizio E.D., Gentile F. (2016). Multiscale Modification of the Conductive PEDOT: PSS Polymer for the Analysis of Biological Mixtures in a Super-hydrophobic Drop. Microelectron. Eng..

[68] Mak C.H., Liao C., Fu Y., Zhang M., Tang C.Y., Tsang Y.H., Chan H.L.W., Yan F. (2015). Highly-sensitive epinephrine sensors based on organic electrochemical transistors with carbon nanomaterial modified gate electrodes. J. Mater. Chem. C.

[69] Ding Y., Tan K., Zhang S., Wang S., Zhang X., Hu P. (2023). Wearable and Recyclable Epinephrine Biosensors Based on Molecular Imprinting Polymer Modified Organic Electrochemical Transistors. Chem. Eng. J..

[70] Hasselmo M.E. (2006). The Role of acetylcholine in learning and memory. Curr. Opin. Neurobiol..

[71] Perry E. (1988). Acetylcholine and Alzheimer’s Disease. Br. J. Psychiatry.

[72] Kergoat L., Piro B., Simon D.T., Pham M.-C., Noël V., Berggren M. (2014). Detection of Glutamate and Acetylcholine with Organic Electrochemical Transistors Based on Conducting Polymer/Platinum Nanoparticle Composites. Adv. Mater..

[73] Fenoy G.E., Bilderling C.v., Knoll W., Azzaroni O., Marmisollé W.A. (2021). PEDOT: Tosylate-Polyamine-Based Organic Electrochemical Transistors for High-Performance Bioelectronics. Adv. Electron. Mater..

[74] Du J., Cullen J.J., Buettner G.R. (2012). Ascorbic Acid: Chemistry, Biology and the Treatment of Cancer. Biochim. Biophys. Acta Bioenerg..

[75] Gkoupidenis P., Schaefer N., Garlan B., Malliaras G.G. (2015). Neuromorphic Functions in PEDOT: PSS Organic Electrochemical Transistors. Adv. Mater..

[76] Liao C., Mak C., Zhang M., Chan H.L.W., Yan F. (2015). Flexible Organic Electrochemical Transistors for Highly Selective Enzyme Biosensors and Used for Saliva Testing. Adv. Mater..

[77] Feng J., Fang Y., Wang C., Chen C., Tang C., Guo Y., Wang L., Yang Y., Zhang K., Wang J. (2023). All-Polymer Fiber Organic Electrochemical Transistor for Chronic Chemical Detection in the Brain. Adv. Funct. Mater..

[78] Zhang L., Wang G., Wu D., Xiong C., Zheng L., Ding Y., Lu H., Zhang G., Qiu L. (2018). Highly Selective and Sensitive Sensor Based on an Organic Electrochemical Transistor for the Detection of Ascorbic Acid. Biosens. Bioelectron..

[79] Becker B.F. (1993). Towards the Physiological Function of Uric Acid. Free Radic. Biol. Med..

[80] Kutzing M.K., Firestein B.L. (2008). Altered Uric Acid Levels and Disease States. J. Pharmacol. Exp. Ther..

[81] Arcangeli D., Gualandi I., Mariani F., Tessarolo M., Ceccardi F., Decataldo F., Melandr F., Tonelli D., Fraboni B., Scavetta E. (2023). Smart Bandaid Integrated with Fully Textile OECT for Uric Acid Real Time Monitoring in Wound Exudate. ACS Sens..

[82] Tao Y., Wang Y., Zhu R., Chen Y., Liu X., Li M., Yang L., Wang Y., Wang D. (2022). Fiber Based Organic Electrochemical Transistor Integrated with Molecularly Imprinted Membrane for Uric Acid Detection. Talanta.

[83] Varki N.M., Varki A. (2007). Diversity in Cell Surface Sialic Acid Presentations: Implications for Biology and Disease. Lab. Investig..

[84] Chen L., Wang N., Wu J., Yan F., Ju H. (2020). Organic Electrochemical Transistor for Sensing of Sialic Acid in Serum Samples. Anal. Chim. Acta.

[85] Thévenot D.R., Toth K., Durst R.A., Wilson G.S. (1999). Electrochemical Biosensors: Recommended Definitions and Classification. Biosens. Bioelectron..

[86] Laborda E., Molina A., Batchelor-McAuley C., Compton R.G. (2018). Individual Detection and Characterization of Non-Electrocatalytic, Redox-Inactive Particles in Solution by using Electrochemistry. ChemElectroChem.

[87] Lichtenstein A.H., Schwab U.S. (2000). Relationship of Dietary Fat to Glucose Metabolism. Atherosclerosis.

[88] Kalsbeek A., Fleur S.l., Flier E. (2014). Circadian control of glucose metabolism. Mol. Metab..

[89] Liao C., Zhang M., Niu L., Zheng Z., Yan F. (2013). Highly Selective and Sensitive Glucose Sensors Based on Organic Electrochemical Transistors with Graphene-modified Gate electrodes. J. Mater. Chem. B.

[90] Liao J., Lin S., Yang Y., Liu K., Du W. (2015). Highly Selective and Sensitive Glucose Sensors Based on Organic Electrochemical Transistors Using TiO_2_ Nanotube Arrays-based Gate Electrodes. Sens. Actuators B Chem..

[91] Zhang X., Liu P., Wang R., Gong Q., Fang C. (2024). Smart Sweat Glucose Detection System Based on Organic Electrochemical Transistor and Near Field Communication. Chin. J. Anal. Chem..

[92] Singh M., Verma N., Garg A.K., Redhu N. (2008). Urea Biosensors. Sens. Actuators B-Chem..

[93] Vanholder R., Gryp T., Glorieux G. (2018). Urea and Chronic Kidney Disease: The Comeback of the Century? (in Uraemia Research). Nephrol. Dial. Transplant..

[94] Berto M., Diacci C., Theuer L., Di Lauro M., Simon D.T., Berggren M., Biscarini F., Beni V., Bortolotti C.A. (2018). Label free urea biosensor based on organic electrochemical transistors. Flex. Print. Electron..

[95] Mohamed S.J., Murugasenapathi N.K., Murugathas T., Gopinath S.C.B., Tamilarasan P. (2024). Chapter 15–Organic Electrochemical Transistor-based Advanced Biosensor for Clinical Diagnosis. Health and Environmental Applications of Biosensing Technologies.

[96] Martinez F.A.C., Balciunas E.M., Salgado J.M., González J.M.D., Converti A., Oliveira R.P.d.S. (2013). Lactic Acid Properties, Applications and Production: A Review. Trends Food Sci. Technol..

[97] Gorbach S.L. (1990). Lactic Acid Bacteria and Human Health. Ann. Med..

[98] Payne M.E., Zamarayeva A., Pister V.I., Yamamoto N.A.D., Arias A.C. (2019). Printed, Flexible Lactate Sensors: Design Considerations Before Performing On-Body Measurements. Sci. Rep..

[99] Khodagholy D., Curto V., Fraser K., Gurfinkel M., Byrne R., Diamond D., Malliaras G., Benito-Lopez F., Owens R. (2012). Organic Electrochemical Transistor Incorporating an Ionogel as Solid State Electolyte for Lactate Sensing. J. Mater. Sci..

[100] Scheiblin G., Aliane A., Strakosas X., Curto V.F., Coppard R., Marchand G., Owens R.M., Mailley P., Malliaras G.G. (2015). Screen-printed Organic Electrochemical Transistors for Metabolite Sensing. MRS Commun..

[101] Ji X., Lau H.Y., Ren X., Peng B., Zhai P., Feng S., Chan P.K.L. (2016). Highly Sensitive Metabolite Biosensor Based on Organic Electrochemical Transistor Integrated with Microfluidic Channel and Poly (N-vinyl-2-pyrrolidone)-Capped Platinum Nanoparticles. Adv. Mater. Technol..

[102] Blanco-López M.C., Gutiérrez-Fernández S., Lobo-Castañón M.J., Miranda-Ordieres A.J., Tuñón-Blanco P. (2004). Electrochemical Sensing with Electrodes Modified with Molecularly Imprinted Polymer Films. Anal. Bioanal. Chem..

[103] Sharma P.S., Dabrowski M., D’Souza F., Kutner W. (2013). Surface Development of Molecularly Imprinted Polymer Films to Enhance Sensing Signals. Trac-Trends Anal. Chem..

[104] Zhang L., Wang G., Xiong C., Zheng L., He J., Ding Y., Lu H., Zhang G., Cho K., Qiu L. (2018). Chirality Detection of Amino Acid Enantiomers by Organic Electrochemical Transistor. Biosens. Bioelectron..

[105] Zhang L., Liu Z., Xiong C., Zheng L., Ding Y., Lu H., Zhang G., Qiu L. (2018). Selective Recognition of Histidine Enantiomers Using Novel Molecularly Imprinted Organic Transistor Sensor. Org. Electron..

[106] O’Connor L., Glynn B. (2010). Recent Advances in the Development of Nucleic Acid Diagnostics. Expert Rev. Med. Devices.

[107] Umek R., Lin S.W., Vielmetter J., Terbrueggen R., Irvine B., Yu C., Kayyem J., Yowanto H., Blackburn G., Farkas D. (2001). Electronic Detection of Nucleic Acids: A Versatile Platform for Molecular Diagnostics. J. Mech. Des..

[108] Kang T., Lu J., Yu T., Long Y., Liu G. (2022). Advances in Nucleic Acid Amplification Techniques (NAATs): COVID-19 Point-of-care Diagnostics as an Example. Biosens. Bioelectron..

[109] Chen C., Song Q., Lu W., Zhang Z., Yu Y., Liu X., He R. (2021). A Sensitive Platform for DNA Detection Based on Organic Electrochemical Transistor and Nucleic Acid Self-assembly Signal Amplification. R. Soc. Chem. Adv..

[110] Song J.J., Lin P., Ruan Y.-F., Zhao W.-W., Wei W.W., Hu J., Ke S.M., Zeng X.R., Xu J.-J., Chen H.-Y. (2018). Organic Photo-Electrochemical Transistor-Based Biosensor: A Proof-of-Concept Study toward Highly Sensitive DNA Detection. Adv. Healthc. Mater..

[111] Li T., Fan Q., Liu T., Zhu X., Zhao J., Li G. (2010). Detection of Breast Cancer Cells Specially and Accurately by an Electrochemical Method. Biosens. Bioelectron..

[112] Liu J., Qin Y., Li D., Wang T., Liu Y., Wang J., Wang E. (2013). Highly Sensitive and Selective Detection of Cancer Cell with a Label-free Electrochemical Cytosensor. Biosens. Bioelectron..

[113] Pan C., Guo M., Nie Z., Xiao X., Yao S. (2009). Aptamer-Based Electrochemical Sensor for Label-Free Recognition and Detection of Cancer Cells. Electroanalysis.

[114] Soda N., Gonzaga Z.J., Chen S., Koo K.M., Nguyen N., Shiddiky M.J.A., Rehm B.H.A. (2021). Bioengineered Polymer Nanobeads for Isolation and Electrochemical Detection of Cancer Biomarkers. ACS Appl. Mater. Interfaces.

[115] Cantor J.R., Sabatini D.M. (2012). Cancer Cell Metabolism: One Hallmark, Many Faces. Cancer Discov..

[116] Madhav N., Shreya S., Pranshu S., Pallavi C., Abbas Z.M. (2016). Tumor Markers: A Diagnostic Tool. Int. J. Oral Maxillofac. Surg..

[117] Alizadeh E., Castle J., Quirk A., Taylor C.D.L., Xu W., Prasad A. (2020). Cellular Morphological Features are Predictive Markers of Cancer Cell State. Comput. Biol. Med..

[118] Sali R., Jiang Y., Attaranzadeh A., Holmes B., Li R. (2024). Morphological Diversity of Cancer Cells Predicts Prognosis across Tumor Types. JNCI—J. Natl. Cancer Inst..

[119] Alitalo K., Keski-oja J., Vaheri A. (1981). Extracellular Matrix Proteins Characterize Human Tumor Cell Lines. Int. J. Cancer.

[120] Tothill I.E. (2009). Biosensors for Cancer Markers Diagnosis. Semin. Cell Dev. Biol..

[121] Fu Y., Wang N., Yang A., Law H.K.-w., Li L., Yan F. (2017). Highly Sensitive Detection of Protein Biomarkers with Organic Electrochemical Transistors. Adv. Mater..

[122] Kailemia M.J., Park D., Lebrilla C.B. (2017). Glycans and Glycoproteins as Specific Biomarkers for Cancer. Anal. Bioanal. Chem..

[123] Wolf G.T., Chretien P.B., Elias E.G., Makuch R.W., Baskies A.M., Spiegel H.E., WeisS J.F. (1979). Serum Glycoproteins in Head and Neck Squamous Carcinoma: Correlations with Tumor Extent, Clinical Tumor Stage, and T-cell Levels During Chemotherapy. Am. J. Surg..

[124] Riley N.M., Wen R.M., Bertozzi C.R., Brooks J.D., Pitteri S.J. (2023). Chapter Four-Measuring the Multifaceted Roles of Mucin-domain Glycoproteins in Cancer. Adv. Cancer Res..

[125] Chen L., Fu Y., Wang N., Yang A., Li Y., Wu J., Ju H., Yan F. (2018). Organic Electrochemical Transistors for the Detection of Cell Surface Glycans. ACS Appl. Mater. Interfaces.

[126] Song Q., Wang W., Liang J., Chen C., Cao Y., Cai B., Chen B., He R. (2023). Fabrication of PEDOT: PSS-based Solution Gated Organic Electrochemical Transistor Array for Cancer Cells Detection. R. Soc. Chem. Adv..

[127] Yeung S.Y., Gu X., Tsang C.M., Tsao S.W.G., Hsing I. (2019). Organic Electrochemical Transistor Array for Monitoring Barrier Integrity of Epithelial Cells Invaded by Nasopharyngeal Carcinoma. Sens. Actuators B-Chem..

[128] Stamey T.A., Yang N., Hay A.R., McNeal J., Freiha F.S., Redwine E.A. (1987). Prostate-Specific Antigen as a Serum Marker for Adenocarcinoma of the Prostate. N. Engl. J. Med..

[129] Lilja H., Ulmert D., Vickers A. (2008). Prostate-specific Antigen and Prostate Cancer: Prediction, Detection and Monitoring. Nat. Rev. Cancer.

[130] Catalona W.J., Smith D.S., Ratliff T.L., Dodds K.M., Coplen D.E., Yuan J.J., Petros J.A., Andriole G.L. (1991). Measurement of Prostate-specific Antigen in Serum as a Screening Test for Prostate Cancer. New Engl. J. Medicine..

[131] Kim D., Lee N., Park J., Park I., Kim J., Cho H.J. (2010). Organic Electrochemical Transistor Based Immunosensor for Prostate Specific Antigen (PSA) Detection Using Gold Nanoparticles for Signal Amplification. Biosens. Bioelectron..

[132] Pantel K., Speicher M. (2016). The Biology of Circulating Tumor Cells. Oncogene.

[133] Ignatiadis M., Lee M., Jeffrey S.S. (2015). Circulating Tumor Cells and Circulating Tumor DNA: Challenges and Opportunities on the Path to Clinical Utility. Clin. Cancer Res..

[134] Martin T.A., Jiang W.G. (2009). Loss of Tight Junction Barrier Function and Its Role in Cancer Metastasis. Biochim. Et Biophys. Acta-Biomembr..

[135] Kominsky S., Argani P., Korz D., Evron E., Raman V., Garrett E., Rein A., Sauter G., Kallioniemi O.-P., Sukumar S. (2003). Loss of the Tight Junction Protein Claudin-7 Correlates with Histological Grade in Both Ductal Carcinoma in Situ and Invasive Ductal Carcinoma of the Breast. Oncogene.

[136] Utoguchi N., Mizuguchi H., Dantakean A., Makimoto H., Wakai Y., Tsutsumi Y., Nakagawa S., Mayumi T. (1996). Effect of Tumour Cell-conditioned Medium on Endothelial Macromolecular Permeability and Its Correlation with Collagen. Br. J. Cancer.

[137] Song Q., Liu H., Wang W., Chen C., Cao Y., Chen B., Cai B., He R. (2024). Carboxyl Graphene modified PEDOT: PSS organic electrochemical transistor for in situ detection of cancer cell morphology. Nanoscale.

[138] Ni Y., Liu J., Han H., Yu Q., Yang L., Xu Z., Jiang C., Liu L., Xu W. (2024). Visualized in-sensor Computing. Nat. Commun..

[139] Ni Y., Yang L., Feng J., Liu J., Sun L., Xu W. (2023). Flexible optoelectronic neural transistors with broadband spectrum sensing and instant electrical processing for multimodal neuromorphic computing. SmartMat.

[140] Nielsen C.B., Giovannitti A., Sbircea D.-T., Bandiello E., Niazi M.R., Hanifi D.A., Sessolo M., Amassian A., Malliaras G.G., Rivnay J. (2016). Molecular Design of Semiconducting Polymers for High-Performance Organic Electrochemical Transistors. J. Am. Chem. Soc..

[141] Chen X., Marks A., Paulsen B.D., Wu R., Rashid R.B., Chen H., Alsufyani M., Rivnay J., McCulloch I. (2021). n-Type Rigid Semiconducting Polymers Bearing Oligo (Ethylene Glycol) Side Chains for High-Performance Organic Electrochemical Transistors. Angew. Chem.-Int. Ed..

[142] Xu S., Liu Y., Lee H., Li W. (2024). Neural Interfaces: Bridging the Brain to the World beyond Healthcare. Exploration.

[143] Xu M., Obodo D., Yadavall V.K. (2019). The Design, Fabrication, and Applications of Flexible Biosensing Devices. Biosens. Bioelectron..

[144] Bocchetta P., Frattini D., Ghosh S., Mohan A.M.V., Kumar Y., Kwon Y. (2020). Soft Materials for Wearable/Flexible Electrochemical Energy Conversion, Storage, and Biosensor Devices. Materials.

[145] Mitcheson P.D. Energy harvesting for human wearable and implantable bio-sensors. Proceedings of the Annual International Conference of the IEEE Engineering in Medicine and Biology.

[146] Premanode B., Toumazou C. (2007). A Novel, Low Power Biosensor for Real Time Monitoring of Creatinine and Urea in Peritoneal Dialysis. Sens. Actuators B Chem..

[147] Lee H., Lee S., Lee W., Yokota T., Fukuda K., Someya T. (2019). Ultrathin Organic Electrochemical Transistor with Nonvolatile and Thin Gel Electrolyte for Long-Term Electrophysiological Monitoring. Adv. Funct. Mater..

[148] Rezali F.A.M., Soin N., Hatta S.F.W.M., Daut M.H.M., Nouxman M.H.A.-H., Hussin H. (2022). Design Strategies and Prospects in Developing Wearable Glucose Monitoring System Using Printable Organic Transistor and Microneedle: A Review. IEEE Sens. J..

[149] Spooren A., Rondou P., Debowska K., Lintermans B., Vermeulen L., Samyn B., Skieterska K., Debyser G., Devreese B., Vanhoenacker P. (2010). Resistance of the Dopamine D4 Receptor to Agonist-induced Internalization and Degradation. Cell. Signal..

[150] Jung H.H., Lee H., Yea J., Jang K.I. (2024). Wearable electrochemical sensors for real-time monitoring in diabetes mellitus and associated complications. Soft Sci..

[151] Ni Y., Wang Y., Xu W. (2021). Recent Process of Flexible Transistor-Structured Memory. Small.

[152] Spanu A., Martines L., Bonfiglio A. (2021). Interfacing Cells with Organic Transistors: A Review of In Vitro and In Vivo Applications. Lab A Chip.

[153] Saha T., Fang J., Mukherjee S., Dickey M.D., Velev O.D. (2021). Wearable Osmotic-Capillary Patch for Prolonged Sweat Harvesting and Sensing. ACS Appl. Mater. Interfaces.

[154] Song Y., Min J., Yu Y., Wang H., Yang Y., Zhang H., Gao W. (2020). Wireless battery-free wearable sweat sensor powered by human motion. Sci. Adv..

[155] Janardhanan J.A., Chen Y., Liu C., Tseng H., Wu P., She J., Hsiao Y., Yu H. (2022). Sensitive Detection of Sweat Cortisol Using an Organic Electrochemical Transistor Featuring Nanostructured Poly(3,4-Ethylenedioxythiophene) Derivatives in the Channel Layer. Anal. Chem..

[156] Yu H., Wei H., Gong J., Han H., Ma M., Wang Y., Xu W. (2021). Evolution of Bio-Inspired Artificial Synapses: Materials, Structures, and Mechanisms. Small.

[157] Zeng M., He Y., Zhang C., Wan Q. (2021). Neuromorphic Devices for Bionic Sensing and Perception. Front. Neurosci..

[158] Barbarossa S., Scutari G. (2007). Bio-Inspired Sensor Network Design. IEEE Signal Process. Mag..

[159] Tata U., Deshmukh S., Chiao J.C., Carter R., Huang H. (2009). Bio-inspired Sensor Skins for Structural Health Monitoring. Smart Mater. Struct..

[160] Yuan Y., Gao R., Wu Q., Fang S., Bu X., Cui Y., Han C., Hu L., Li X., Wang X. (2023). Artificial Leaky Integrate-and-Fire Sensory Neuron for In-Sensor Computing Neuromorphic Perception at the Edge. ACS Sens..

[161] Wu M., Yao K., Huang N., Li H., Zhou J., Shi R., Li J., Huang X., Li J., Jia H. (2023). Ultrathin, Soft, Bioresorbable Organic Electrochemical Transistors for Transient Spatiotemporal Mapping of Brain Activity. Adv. Sci..

[162] Wang Y., Liu S., Wang H., Zhao Y., Zhang X.-D. (2022). Neuron Devices: Emerging Prospects in Neural Interfaces and Recognition. Microsyst. Nanoeng..

[163] Ni Y., Liu L., Feng J., Yang L., Xu W. (2023). Flexible Organic Artificial Synapse with Ultrashort-term Plasticity for Tunable Time-frequency Signal Processing. Chin. Chem. Lett..

